# Clinical Findings, Treatment Recommendations, and Outcome in Dogs Following Ingestion of a Single Unintended Dose of Trilostane: 403 Cases (2008–2023)

**DOI:** 10.1111/vec.70112

**Published:** 2026-05-21

**Authors:** Lindsey Summers, Jenny Schuett, Laura Stern

**Affiliations:** ^1^ Department of Internal Medicine MSPCA Angell Animal Medical Center Boston Massachusetts USA; ^2^ ASPCA Animal Poison Control Center Champaign Illinois USA

**Keywords:** canine, exposure, iatrogenic hypoadrenocorticism, intoxication

## Abstract

**Objective:**

To characterize the clinical signs and outcomes in dogs following ingestion of a single, unintended dose of trilostane. The secondary objective was to describe treatment recommendations given for this population.

**Design:**

Retrospective evaluation of cases of canine trilostane exposure from February 2008 to August 2023.

**Setting:**

Private, specialty toxicology consultation service.

**Animals:**

Four hundred three cases were selected from the American Society for the Prevention of Cruelty to Animals (ASPCA) Animal Poison Control Center AnTox database using a nonstratified sampling technique. Data included patient demographics, history, therapeutic use of trilostane, exposure history, diagnostic findings, treatment recommendations, and outcome.

**Interventions:**

None.

**Methods and Main Results:**

The most common cause of trilostane exposure was ingestion of another dog's prescription (248/403 [61.5%]), followed by ingestion of extra or excessive dose(s) of the dog's own prescription (147/403 [36.5%]). Forty‐one dogs (41/403 [10.2%]) had clinical signs attributable to trilostane exposure. Median onset of clinical signs after exposure was 2.5 h (range: 0.25–72 h). The most frequently reported clinical signs were vomiting (17/41 [41.5%]), lethargy (15/41 [36.6%]), and diarrhea (7/41 [17.0%]). Four cases (4/403 [0.99%]) were reported to require veterinary treatment for trilostane toxicosis. Of the cases with follow‐up from the ASPCA (200/403 [49.6%]), most dogs (188/200 [93.5%]) developed no clinical signs from acute trilostane exposure. The median exposure dosage (13.2 mg/kg) in the 12 dogs that developed clinical signs was significantly higher than in those that did not (3.3 mg/kg).

**Conclusions:**

The current study suggests that dogs ingesting a single, unintended dose of trilostane are likely to experience mild, self‐limiting signs. The need for extensive hospitalization or aggressive medical care is unlikely, even with high single doses that exceed the published and Food and Drug Administration‐approved therapeutic dosage.

**Abbreviations**
APCC: Animal Poison Control Center
ASPCA: American Society for the Prevention of Cruelty to Animals
FDA: Food and Drug Administration
IQR: interquartile range

## 1 Introduction

**FIGURE 1:**
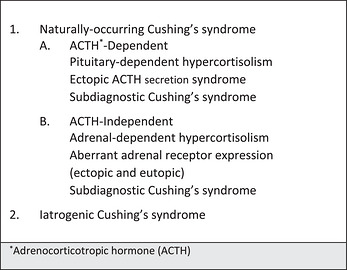
The ALIVE Project definitions proposed by the European Society of Veterinary Endocrinology for classification of hypercortisolism [1].

Canine Cushing's syndrome, or hypercortisolism, encompasses a range of clinical syndromes characterized by chronic and excessive endogenous or exogenous glucocorticoid exposure. Hypercortisolism is further subcategorized by the underlying pathogenesis, including naturally occurring or iatrogenic Cushing's syndrome (Figure 1) [1]. The term hyperadrenocorticism refers to an overproduction of cortisol by the adrenal glands. Clinical signs of canine hypercortisolism, including but not limited to polyuria with compensatory polydipsia, polyphagia, panting, weight gain, muscle weakness, and hair loss, result from the combined gluconeogenic, immunosuppressive, anti‐inflammatory, protein catabolic, and lipolytic effects of glucocorticoids [1]. Treatment of choice for hypercortisolism depends on several factors, including the underlying pathogenesis, severity of disease, presence of comorbidities, treatment efficacy, clinician–client predilections, and potential side effects.

Trilostane is an oral adrenocortical suppressant labeled for use in dogs for the chronic treatment of pituitary‐dependent and adrenocortical‐dependent hyperadrenocorticism [2]. Proposed use of trilostane for the treatment of canine hyperadrenocorticism was published in 1998 and approved by the US Food and Drug Administration (FDA) in 2008 [2, 3]. Trilostane acts as a reversible competitive inhibitor of 3‐β‐hydroxysteroid dehydrogenase and limits the synthesis of endogenous hormones such as cortisol, aldosterone, and adrenal androgens [4]. Several animal safety and efficacy studies have shown trilostane to be an effective treatment for dogs with spontaneous hypercortisolism [2, 5]. Although dogs have been shown to tolerate trilostane administration relatively well, adverse effects have been reported, such as lethargy, vomiting, diarrhea, and mild electrolyte abnormalities (e.g., hyponatremia, hyperkalemia) [4]. These adverse effects are typically mild in nature and self‐limiting [4]; however, meaningful clinical consequences, such as lifelong hypoadrenocorticism, have been reported in some individuals [6].

Studies in dogs with a history of chronic or sequential administration of therapeutic dosages of trilostane have reported both mild and more impactful adverse effects [2, 5, 6, 7, 8], but literature regarding the clinical impact of acute exposures is limited. The purpose of the current study was to report the clinical signs, treatments, and outcomes in dogs following ingestion of a single, unintended dose of trilostane.

## 2 Materials and Methods

Canine cases with a history of trilostane exposure established with the American Society for the Prevention of Cruelty to Animals (ASPCA) Animal Poison Control Center (APCC) AnTox program from February 2008 to August 2023 were identified. AnTox is a comprehensive clinical animal toxicology data collection and retrieval system that facilitates identification of toxic effects of various substances in animals [3].

Cases of potential exposure to trilostane were identified via a nonstratified collection method. Cases of noncanine species were excluded. Dogs with the potential for concurrent exposure to additional agents (e.g., over‐the‐counter medications, supplements, household products) were excluded. Dogs with repeated exposures to trilostane beyond a single extra administered therapeutic dose were also omitted. Finally, cases were excluded if data were incomplete, or if the dog had clinical signs deemed by the consulting ASPCA APCC veterinarian or veterinary toxicologist as unlikely related to trilostane exposure due to the presence of other cause for the clinical signs or the signs’ onset or duration.

Dogs with underlying health conditions in addition to hypercortisolism were included in the study. These comorbidities included diabetes mellitus, hypothyroidism, orthopedic diseases, hepatic insufficiency, gastrointestinal conditions, urinary tract disease, cognitive dysfunction syndrome, seizures, cardiac disease, systemic hypertension, and history of pancreatitis. Dogs prescribed other medications besides trilostane, such as insulin therapy, were included in the study. The administration timing and dosages of any other medications were not evaluated.

### 2.1 Statistical Analysis

Descriptive statistics were used for the patient demographic data. Mann–Whitney *U*‐ and *χ* ^2^ tests were used to evaluate differences between categorical variables (e.g., trilostane exposure dosages between clinically affected and asymptomatic cases) that were not normally distributed. Continuous variables, such as age and trilostane dosage, were summarized with medians and ranges (including interquartile range [IQR]) rather than means and SDs due to the nonnormal distribution of the data. Medians provide a robust measure of central tendency in the presence of skewed data or outliers and ensure a more accurate representation of the typical values observed in the current study. Significance was set at *p* < 0.05.

## 3 Results

### 3.1 Population Characteristics

**FIGURE 2:**
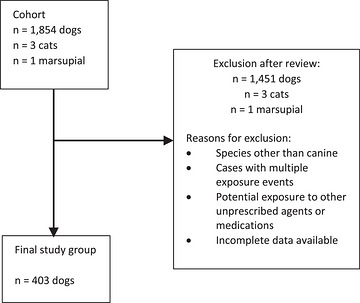
Consort‐style diagram showing the screening, exclusion, and enrollment of dogs with a single, unintended ingestion of trilostane in the Animal Poison Control Center AnTox program.

Reported cases of trilostane exposure were retrieved from the AnTox database, of which 403 fit the inclusion criteria (Table 1; Figure 2). One hundred eighty‐six dogs (46.0%) were neutered females, 166 (41.1%) were neutered males, 29 (7.2%) were intact males, and 23 (5.7%) were intact females. The median age of dogs in the study was 8.5 years old (range: 0.2–20 years).

**TABLE 1:** Characteristics of 403 dogs with a single, unintended ingestion of trilostane.

Case characteristics
Breed
Sex
Age
Body weight
Exposure‐related data
Type of incident
(Estimated) dose ingested
Calculated dosage (mg/kg)
Medical history before ingestion
Clinical signs
Diagnosis
Clinical course and treatment
Time to onset of clinical signs following exposure
Clinical signs observed
Clinicopathologic data
Treatment recommended via Animal Poison Control Center
Follow‐up information

Two hundred forty‐eight of 403 dogs (61.5%) had ingested medication from another dog's trilostane prescription, while 147 dogs (36.5%) had voluntarily ingested excess dose(s) of their own prescription. Six of 403 dogs (1.5%) were inadvertently overdosed by owners. One dog was unintentionally dispensed trilostane instead of another unknown medication. Another dog was prescribed 5 mg of trilostane (0.9 mg/kg) daily, but 60 mg (10.7 mg/kg) was inadvertently dispensed and was administered one time.

Canine hypercortisolism had been diagnosed previously in 154 of 403 dogs (38.2%). Of these 154 cases, most dogs (147/154 [95.5%]) had ingested their own trilostane. In the current study, osteoarthritis, unspecified hepatopathy, unspecified cardiac conditions, and diabetes mellitus were the most commonly reported concurrent comorbidities in dogs with Cushing's syndrome.

### 3.2 Clinical Signs and Exposure Doses

**FIGURE 3:**
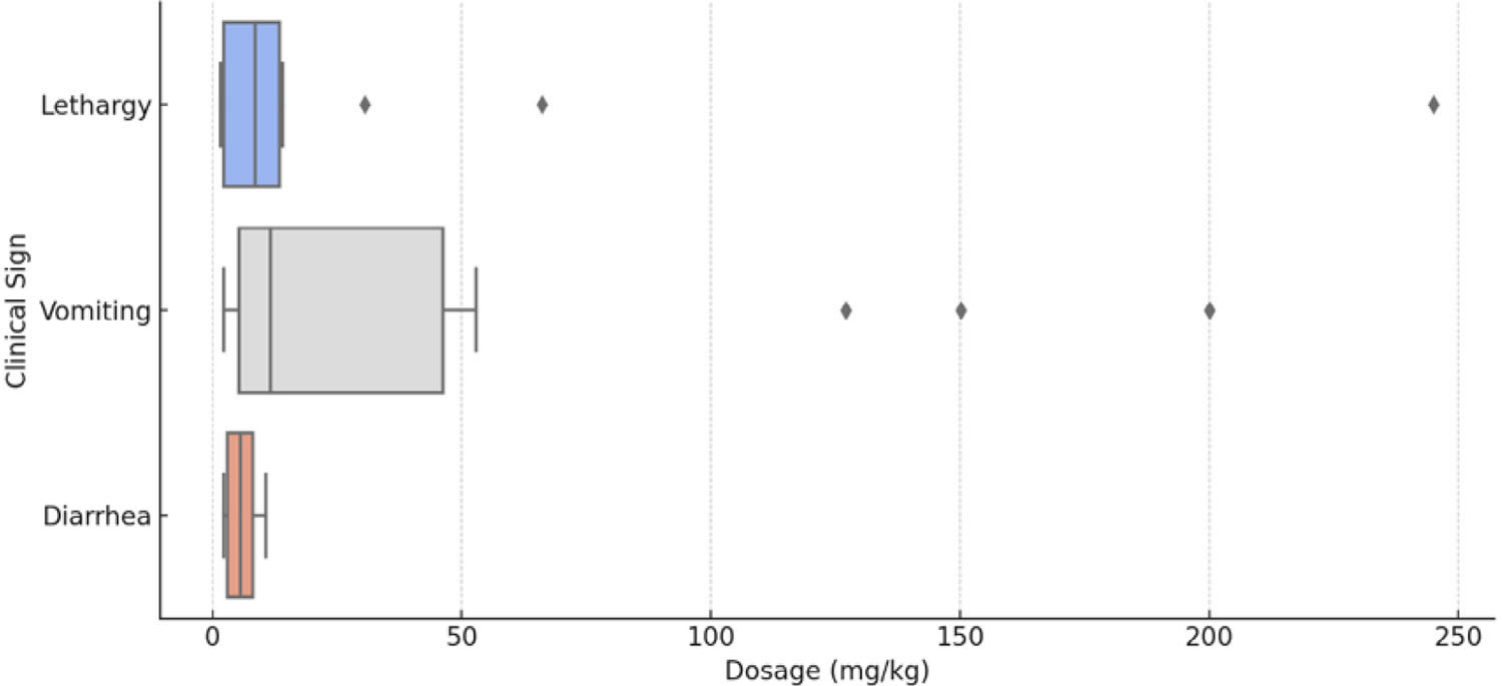
Boxplot representing the top three most reported clinical signs in dogs with a single, unintended ingestion of trilostane and their respective trilostane dosages. The box represents data from the first to third quartile, while the median is represented by the horizontal line within each box. The whiskers represent the range. Dots represent individual outliers.

Exposure dose was calculated at the time of case establishment with the ASPCA in all 403 dogs (Table 2). Forty‐one dogs (10.2%) had active clinical signs reported after ingestion of trilostane, the most common of which were vomiting (17/41 [41.5%]), lethargy (15/41 [36.6%]), and diarrhea (7/41 [17.0%]). Other clinical signs included ataxia, restlessness, hypersalivation, inappetence, urticaria, and trembling, representing a small percentage (≤5%) of the study population. The median onset of clinical signs for these 41 cases was 2.5 h following exposure (range: 0.25–72 h). The median dosage of trilostane exposure for these 41 cases was 10 mg/kg (range: 1.6–245 mg/kg). The median ingested dose for the remaining 362 cases was 2.94 mg/kg (range: 0.2–202.5 mg/kg; *U* = 11,216.5; *p* < 0.05). The more severe clinical signs (e.g., vomiting) were associated with higher dosages of trilostane; however, some of the most commonly reported signs (e.g., lethargy, diarrhea) were associated with lower dosages (Figure 3). The median age of dogs that developed clinical signs was 10.0 years (IQR: 7.25–12.375; range: 0.1–20 years), whereas the median age for those without clinical signs was 8.0 years (IQR: 2.9–12.0; range: 0.1–17 years; *U* = 8992.5; *p* = 0.0563).

**TABLE 2:** Features of 403 dogs after a single, unintentional ingestion of trilostane.

Dosage, mg/kg	Number of dogs	Clinical signs and number of dogs	Follow‐up (NS24^a^/unknown)
<1.0	55	—	34/21
1.0–4.9	184	Vomiting (*n* = 4)	87/97
		Diarrhea (*n* = 3)	
		Lethargy (*n* = 6)	
5.0–9.9	65	Vomiting (*n* = 3)	31/34
		Diarrhea (*n* = 3)	
		Lethargy (*n* = 4)	
10–50	69	Vomiting (*n* = 5)	35/33^b^
		Diarrhea (*n* = 1)	
		Lethargy (*n* = 4)	
>50	30	Vomiting (*n* = 6)	12/18
		Lethargy (*n* = 2)	

*Note*: The clinical signs reported were observed at the time of case establishment with the Animal Poison Control Center.

^a^ NS24: No clinical signs after 24 h of exposure to trilostane.

^b^ One case in this group was euthanized and is thus not counted here.

Clinicopathologic data within 24 h of unintended trilostane exposure were reported in nine of the 403 cases, including mild hyperkalemia (seven dogs, range: 5.1–5.5 mmol/L [5.1–5.5 mEq/L]), hyponatremia (two dogs, range: 134–144 mmol/L [134–144 mEq/L]), monocytosis (one dog, value not recorded), and hypercalcemia (one dog, value not recorded). A *χ* ^2^ test identified an association between the availability of clinicopathologic data and the presence of clinical signs in dogs with a single, unintended ingestion of trilostane (*χ* ^2^ = 8.12; *p* = 0.0044). Additionally, dogs with reported clinicopathologic data had ingested significantly higher trilostane dosages (median: 56.3 mg/kg, IQR: 6.56–154.0 mg/kg; range: 2.2–245 mg/kg) compared with dogs without clinicopathologic data reported (median: 3.3 mg/kg, IQR: 1.47–9.44 mg/kg; range: 0.2–220.5 mg/kg; *U* = 3647.0; *p* < 0.001).

### 3.3 Treatment Recommendations and Outcome

Treatment and monitoring recommendations were presented by the ASPCA APCC to the owners of all dogs in the study. Veterinary care was recommended in 204 of 403 dogs (50.6%), while home monitoring was recommended in 199 (49.4%). Seventy‐seven of the 204 dogs (37.7%) for which veterinary care was recommended ingested trilostane doses exceeding the labeled dosage (2.2–6.7 mg/kg/day) [2, 4]. The ASPCA representative recommended veterinary care at the time of case establishment to owners of all 41 dogs that developed clinical signs. The median dosage of trilostane exposure in the 199 cases with recommendations for home monitoring was 2.8 mg/kg (range: 0.28–220.5 mg/kg). Fifty‐five of the 199 dogs (27.6%) for which home monitoring was recommended had ingested a dose that exceeded the labeled dosage; however, none of these dogs reportedly developed clinical signs.

Of the 403 dogs studied, four (0.99%) reportedly received veterinary care. Two dogs received outpatient therapies such as oral antihistamine, subcutaneous fluids, and subcutaneous antiemetic medication. One case (0.25%) required 24 h of hospitalization, while another (0.25%) was euthanized after cardiopulmonary arrest.

Two hundred dogs (49.6%) had follow‐up information available 24 h after unintended trilostane exposure. Most of these dogs (188/200 [94%]) had not developed clinical signs. The remaining 12 dogs had developed clinical signs; however, signs did not persist beyond 24 h. The median trilostane dosage in the 12 dogs with 24‐h follow‐up that developed clinical signs was 13.2 mg/kg (IQR: 7.43–32.2 mg/kg; range: 2.2–202 mg/kg), which was higher than that of the 188 dogs with 24‐h follow up that did not develop clinical signs (median: 3.3 mg/kg; IQR: 1.46–9.42 mg/kg; range: 0.2–220.5 mg/kg; *U* = 367.0; *p* < 0.011).

There was no difference in the development of clinical signs between dogs that ingested a trilostane dosage below/within (*n* = 271 dogs) or above (*n* = 132) the labeled dose (*χ* ^2^ = 0.27; *p* = 0.606).

## 4 Discussion

This is one of the first studies to assess the clinical signs in dogs having ingested a single unintended dose of trilostane. Common clinical signs included vomiting, lethargy, and diarrhea. According to the trilostane product label, the most common adverse effects are similar to the clinical signs observed in the current study [2]. During clinical trials of trilostane before product launch, two dogs were reported to experience impactful adverse reactions, including adrenal necrosis/rupture and hypoadrenocorticism, respectively [2]. Although the doses administered in prerelease trials were not available for comparison with dogs in the current study, the clinical trial dogs exhibited adverse signs after continuous administration of trilostane (i.e., weeks). The dogs in the current study were exposed to a single unintended oral dose of trilostane.

Several variables including body weight, frequency of administration, client compliance, response to therapy, and diagnostic testing results often determine the appropriate dosing of trilostane for the treatment of canine hypercortisolism. Several dose recommendations have been published for clinical reference.^1^ Since the drug's approval in the United States, studies regarding the dose of trilostane in dogs with hypercortisolism suggest that there is wide variation in the dose required to control clinical signs [9]. Based on some of these studies, the Plumb's Veterinary Drug formulary^2^ has suggested the FDA labeling may be too high for therapeutic use in canine hypercortisolism. In the current study, dogs were exposed to a wide range of ingested doses of trilostane (0.2–245 mg/kg). No difference in outcome was found between the 132 dogs in this study that were exposed to doses exceeding the FDA‐recommended therapeutic range of 2.2–6.7 mg/kg/day and dogs that ingested a dose at or below the labeled dose.

At the time of initial case establishment with the ASPCA, most of the dogs (362/404 [89.8%]) in the current study had no reports of clinical signs. Ingestion of higher dosages of trilostane was associated with development of clinical signs, as expected. Even with the development of clinical signs, no dogs with follow‐up at 24 h had persistent clinical signs. This suggests that even at high exposure dosages, clinical signs appear to be mild and self‐limiting in most cases.

A few dogs in the current study had more significant clinical signs, such as ataxia or trembling, associated with trilostane exposure. One of the dogs (0.25%) was reported to experience cardiopulmonary arrest and was subsequently euthanized. It is unknown whether trilostane exposure (13.2 mg/kg dose) was the culprit of this dog's outcome because there was no access to the complete medical record. Moreover, it was reported to the ASPCA that the dog had comorbidities, including an unspecified cardiac condition. Despite this single case, the current study largely demonstrated that, even with high dosages, it is unlikely for dogs with ingestion of a single, unintended dose of trilostane to exhibit severe or prolonged side effects.

Persistent hypocortisolemia has been reported in some dogs as an adverse effect of repeated dosing with trilostane [4, 5, 10, 11]. In a previous report, three doses of trilostane (5 mg/kg; 60 mg daily) were administered to a dog before presenting to an emergency facility with gastrointestinal signs, including vomiting and diarrhea. After supportive treatment (including prednisone), the dog was discharged from the hospital [10]. Another paper reported a dog that had received 13 consecutive doses of trilostane ranging from 2 to 4 mg/kg daily. This dog was hospitalized and given supportive therapy after an ACTH stimulation test confirmed iatrogenic hypoadrenocorticism [11]. In the current study, 161 dogs (40.0%) were exposed to a higher single, unintentional oral dose than the dogs in these previous reports of dogs with iatrogenic hypoadrenocorticism resulting from repeated dosages. Of the cases that had follow‐up information, no clinical signs were reported beyond 24 h of the exposure.

A small number of cases in the current study (9/403 [2.2%]) had clinicopathologic data provided to the ASPCA at the time of initial case establishment. There was an association between a report of clinical signs and the availability of clinicopathologic data, probably because veterinarians are more likely to perform laboratory testing in sicker animals. Of these nine cases, eight dogs recovered (88.9%) with no further clinical signs 24 h following exposure; however, the final dog was euthanized after experiencing cardiopulmonary arrest. The most common electrolyte abnormalities in these nine cases were hyperkalemia (range: 5.1–5.5 mmol/L [5.1–5.5 mEq/L]) and hyponatremia (range: 134–144 mmol/L [134–144 mEq/L]). These findings may suggest transient iatrogenic hypoadrenocorticism, but definitive testing was not reported to the ASPCA in any case.

Trilostane is rapidly absorbed after oral administration in dogs, with peak plasma levels 1.5–2 h following ingestion^b^. One prospective study in dogs with pituitary‐dependent hypercortisolism assessed the plasma concentrations of cortisol, endogenous ACTH, aldosterone, and electrolytes after administration of trilostane. The dogs were given trilostane doses between 3.3 and 5.2 mg/kg/day for 30 consecutive days. Baseline cortisol concentrations decreased significantly 2–4 h after trilostane administration but gradually returned to preadministration concentrations before the next daily dose was given [12].

To diagnose hypoadrenocorticism, the gold standard is to assess the functional capacity of the adrenal axis. Typically, an ACTH stimulation test is performed to evaluate the ability of the adrenal glands to produce cortisol. Given that cortisol concentrations have been shown to be the lowest 2–4 h after trilostane administration, an ACTH stimulation test could be used to monitor for transient iatrogenic hypoadrenocorticism in dogs with suspected trilostane intoxication; however, the need to pursue this testing appears to be low considering even dogs with laboratory abnormalities most often had self‐limiting signs. Serial ACTH stimulation tests could be considered to assess for persistent iatrogenic hypoadrenocorticism, assuming a full systemic assessment, including hematologic data and biochemical analyses, corroborated the suspicion.

In a previous study assessing serial electrolyte concentrations in dogs given therapeutic doses of trilostane for 30 days, plasma sodium concentrations remained within the reference interval throughout the day in the majority of dogs (5/9 [55.6%]) [12]. The same study found the sodium‐to‐potassium ratio was most often >27, and thus the study suggested that the electrolyte abnormalities typically seen in hypoadrenocorticism are uncommon in dogs with pituitary‐dependent hypercortisolism treated chronically with trilostane [12]. Even the few dogs with reported electrolyte abnormalities in the current study generally had resolution of clinical signs within 24 h, though serial electrolyte concentrations were not reported and are thus unknown.

Given the retrospective nature of the current study, there were several limitations, including cases that lacked clinicopathologic data or adrenal axis testing to detect subclinical iatrogenic hypoadrenocorticism. There was also limited follow‐up information available, with fewer than half having data available to 24 h. Some dogs in the study population were included despite having comorbidities and daily concurrent medication administrations, such as insulin. The impact of comorbidities and concurrent medication administration on evolution of clinical signs in dogs with a single, unintended ingestion of trilostane was not evaluated.

In conclusion, dogs with a single, unintended ingestion of trilostane are likely to have mild, self‐limiting clinical signs expected to include vomiting, lethargy, and diarrhea. All dogs exhibiting clinical signs of trilostane toxicosis should be managed on an individual basis; however, the need for extensive hospitalization or therapies is unlikely, even with reasonably large single exposure. Studies that evaluate for long‐term effects of a single, unintended ingestion of trilostane are warranted.

## Author Contributions

**Lindsey Summers**: conceptualization, investigation, writing – original draft, methodology, writing – review and editing, visualization, data curation, resources, formal analysis. **Jenny Schuett**: data curation, resources, formal analysis, software. **Laura Stern**: funding acquisition, project administration, supervision, writing – review and editing, data curation.

## Ethics Statement

The authors confirm that the ethical polices of the journal have been adhered to. No ethical approval was required as this is a review article with no original research data.

## Conflicts of Interest

The authors declare no conflicts of interest.

## Endnotes

1 Vetoryl (tilostane). Capsules [Package Insert]. Dechra Veterinary Products, Overland Park, KS.

2 Plumb DC. Trilostane. In: Plumb's veterinary drug handbook. 9th ed. Ames (IA): Blackwell; 2018.
